# Formation of a 3D Particle Array Actuated by Ultrasonic Traveling Waves in a Regular Polygon Resonator

**DOI:** 10.3390/mi13112003

**Published:** 2022-11-17

**Authors:** Fei Wan, Kai Xu, Hongcheng Wang, Haihao Xu, A’long Huang, Zihao Bai, Linan Zhang, Liqun Wu

**Affiliations:** School of Mechanical Engineering, Hangzhou Dianzi University, Hangzhou 310018, China

**Keywords:** microfluidics, particle pattern, acoustic radiation force, particle–fluid flow

## Abstract

Acoustic radiation forces have been extensively studied regarding static particles, cell patterning, and dynamic transportation. Compared with standing wave manipulation, traveling wave manipulation can be more easily modulated in real time and has no matching requirement between the size of the resonant cavity and the sound frequency. In this work, we present an efficient, multi-layer microparticle pattern technique in a 3D polygon cavity with a traveling bulk acoustic wave. There are two types of excitation modes: the interval excitation mode (IEM) and the adjacent excitation mode (AEM). We conducted theoretical and simulation analyses, and our results show that both of these modes can form particle arrays in the resonant cavity, which is in accordance with the experimental results. The array spacings in the IEM and AEM were about 0.8 mm and 1.3 mm, respectively, while the acoustic frequency was 1MHz. Double-layer particle patterns were arrayed by a double in the resonant cavity. The spacing between the two layers was set at 3.0 mm. The line spacings were about 0.4 mm in both layers. The line width was 0.2 mm, which was larger than the single layer. The results show that ultrasonic traveling waves are a feasible method to manipulate particles and cells that form 3D patterns in particle–fluid flows.

## 1. Introduction

The contactless manipulation of fine particles or cells in particle–fluid flows has important applications in many fields, such as three-dimensional printing [1], microfluidic-based cell analysis [2,3], medical engineering [4], material engineering, etc. Compared with optical [5], magnetic [6], electric [7], and other manipulation methods, acoustic technology [8,9] has the advantage of being suitable for any material with variable properties.

In the process of cell manipulation using the acoustic method, a high-frequency soundwave (typically 1–20 MHz) is passed through the flow area of interest using a transducer, and an acoustic radiation force is produced to move nanometer-sized objects. The acoustic waves [10] include surface acoustic waves (SAWs) [11], which are excited by interdigital transducers (IDTs), and bulk acoustic waves (BAWs), which are excited by stacked piezoelectric ceramics. Both of the above can generate standing waves in particle–fluid flows. As for standing SAWs, one-dimensional and two-dimensional interference patterns can be created by using two and four IDT sets, respectively. David J. Collins et al. [12] introduced a new method for patterning multiple, spatially separated single particles and cells using high-frequency acoustic fields with one cell per acoustic well. They characterized and demonstrated patterning for a range of particle sizes, as well as capturing and patterning cells, including human lymphocytes and red blood cells. Yongqing Fu et al. [13] designed flexible SAW devices to significantly change the distribution of particle pattern lines and patterned yeast cells. BAWs use piezoelectric transducers to convert electrical signals into mechanical waves. Acoustic waves reflected from the reflection layer form standing waves and distribute pressure in the fluid. The distance between the piezoelectric transducer and the reflector should be an integer multiple of a sound half-wavelength. A basic configuration for acoustophoresis in bulk acoustic wave devices consists of a fluidic channel with two parallel, opposing walls. Ivo Leibacher et al. [14] employed a BAW ultrasonic standing wave between two opposing silicon walls, both of which acted as an acoustic wave to concentrate microparticles in the channel. For better manipulation, there is strong demand for a way to dynamically manipulate particle patterns, a subject that has not yet been fully investigated. Yongmao Pei. et al. [15] proposed parametric bulk acoustic waves to realize deformable dynamic patterning in multiple particles. Multiple particles were trapped on deformable oscillation lines; then, the lines bent and elongated periodically. Rohan Shirwaiker et al. [16] constructed a hexagonal resonant cavity and used ultrasonic standing bulk acoustic waves to organize cells into controllable anisotropic patterns within viscous bio-inks, while maintaining high cell viability.

To overcome the fact that standing waves are typically generated from normal incidences, Xuefeng Zhu et al. [17] developed a method of ultrasonic retroreflection tweezing via metagratings to obtain the exact relationship between the metagrating geometries and the retroreflection angles in a wide range. They also proposed using an ultrasonic meta-lens to generate super-oscillation acoustic wave-packets with different spatial momenta and then superimposing them on a diffraction limit-broken spot, visually represented by a ring-shaped trap made of tiny particles. This method is suitable for advanced acoustic imaging, biomedical applications, and versatile, far-field ultrasound control [18].

For standing waves to operate microparticles, the distance between the sound source and the reflector should be an integer multiple of the half-wavelength of the ultrasound [19]. It is not convenient to vary the particle pattern in the resonant cavity. As a potential method of overcoming the above problem, traveling waves [20] can form arbitrary pressure nodes in 3D space by controlling the phase patterns of acoustic waves. Jing Sung et al. [21] managed to control the movement of 5 μm PS particles in a polydimethylsiloxane (PDMS) microfluidic channel using tunable traveling surface acoustic waves (TSAWs) produced by a pair of slanted, interdigitated transducers (SIDTs). Compared with standing wave manipulation, traveling wave manipulation can be more easily modulated in real time, and there is no requirement to match the size of the resonant cavity with the sound frequency.

In this work, we present an efficient, microparticle manipulation technique in a 3D polygon cavity with a traveling bulk acoustic wave.

## 2. Materials and Methods

### 2.1. Experiment Setup

**Figure 1:**
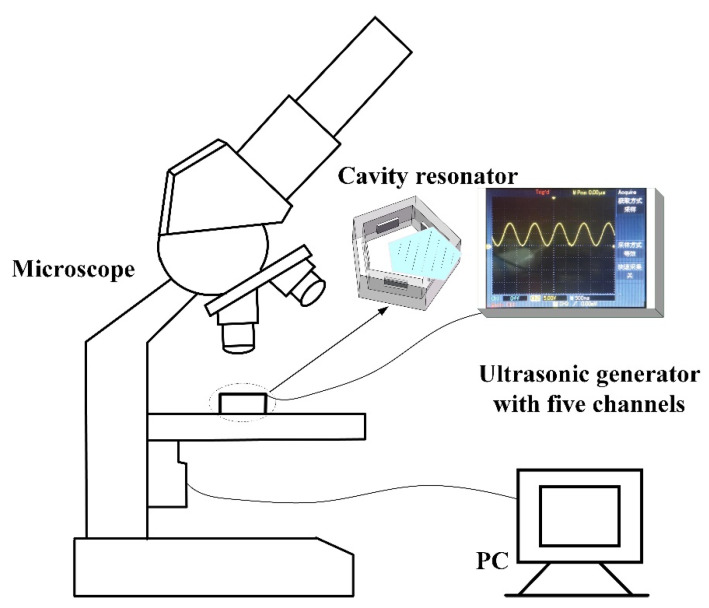
Ultrasonic array, sound pressure, micro-potential, well cell, non-contact manipulation experimental system.

The ultrasonic array, sound pressure, micro-potential, well cell, non-contact manipulation experimental system consists of a microscopic observation system and an acoustic radiation force manipulation system. As shown in Figure 1, the microscopic observation system is composed of a microscope (CX30, Olympus) and an industrial camera. The acoustic control system patterns the particles. The observation system’s microscope observes the experimental particle arrangement process. The microscope is backlit, and the magnification is 100–1200 times. The industrial camera is connected to the microscope to take pictures of the microscopic image. The industrial camera used has a frame rate of 2.91 beats per second and is used to record the movement of the particles in the liquid.

Pentagonal acoustic devices and ultrasonic generation systems constitute acoustic radiation force control systems. The ultrasonic generator with five channels includes a driving board, a signal generator, an operational amplifier, a positive and negative power module, and a battery. The signal generator is from the Conway AD98 model generator, with a maximum output frequency of 30 MHz, an output voltage of 0.53 Vpp, and an output current of 20 mA. The generator can actuate five ultrasonic transducers. The power amplifier is a TSH3001 op amp with a wide gain of 42 MHz, a maximum output voltage of 14 V, and a maximum gain of 100×, which can be adjusted using a rotary button. The voltage amplitude of the sinewave generated by the general signal generation module is 250 mv, and it is impossible to drive the lead zirconate titanate PZT piezoelectric ceramic on the inner wall of the resonator. To obtain the appropriate voltage value and calculate the maximum gain multiple of the system, we use an oscilloscope to test the circuit waveform signal, which is a cosine waveform.

### 2.2. Structure of Pentagonal Resonator

**Figure 2:**
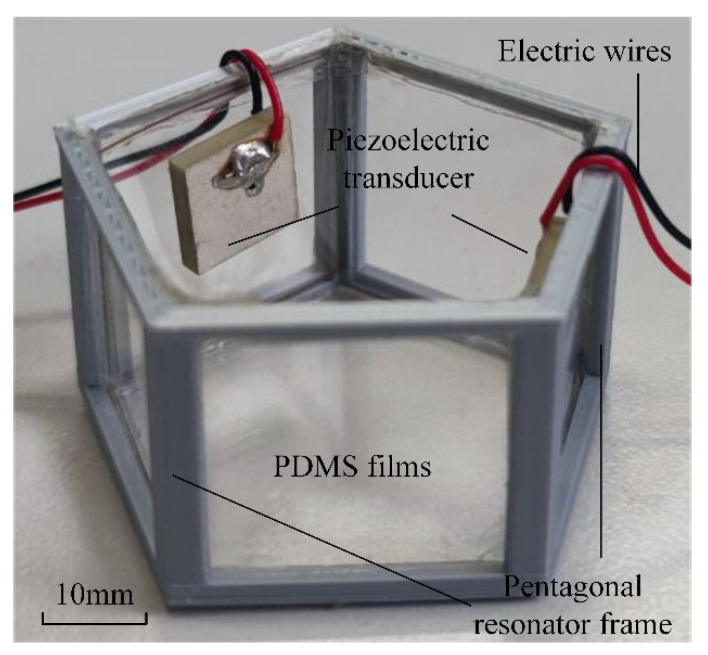
Structure of the pentagonal resonator.

A cavity resonator with pentagonal walls is used as the acoustic generator carrier. The entire structure of the generator is a pentagonal column structure, as shown in Figure 2.

Photosensitive resin is the primary material used to print the pentagonal resonator structure, including the pentagonal profile and column support frame. The specific production process is as follows: The photosensitive resin is printed using 3D printing technology to create a pentagonal skeletonized outer-boundary column frame; the pentagonal outer circle radius is 10 mm, the thickness is 2 mm, and the column height is 12 mm. Additionally, we leave a 2 mm aperture in the center of the bottom of the pentagonal bracket. The whole intersurface of the 3D-printed pentagonal resonator frame is covered with a complete piece of PDMS film with a thickness of 0.2 mm (ZHK-1-02, Hangzhou Bold Advanced Materials Co., Ltd., Hangzhou, China). A pentagonal PMMA plate is pressed on the bottom of the resonator to fix the PDMS film in place. At the same time, the film is tensioned, and after tensioning, film glue is applied to the boundary of the pentagonal frame to keep it in place.

On the side of the device is a vibration excitation element. Its material is a lead zirconium titanate (PZT) piezoelectric ceramic, the parameters of which are shown in Table 1. In addition, piezoelectric ceramics must be in contact with water during the experiment; however, they are not insulators and need to be waterproofed. Therefore, epoxy resin is used to coat the piezoelectric ceramic surface, and then again to bond the pentagonal inner wall to affix it.

There are two types of excitation modes: the adjacent excitation mode (AEM) and the interval excitation mode (IEM). For the AEM mode, the piezoelectric transducers are set on the adjacent boundaries of the resonator. For the IEM mode, the piezoelectric transducers are set on the interval boundaries of the resonator.

### 2.3. Formation of Acoustic Pressure Potential Wells

#### 2.3.1. Theory

We use a traveling wave coupling excited by a biaxial sound source to generate a sound field, based on the principle of wave interference. Suppose there are two soundwave columns in space: the expressions are as follows in Formulas (1) and (2).

(1) $$p_{1}(r,t)=p_{1}\left(r,\omega_{1}\right)\exp\left[-i\left(\omega_{1}t-\varphi_{1}\right)\right]$$

(2) $$p_{2}(r,t)=p_{2}\left(r,\omega_{2}\right)\exp\left[-i\left(\omega_{2}t-\varphi_{2}\right)\right]$$

*ω*_1_ and *ω*_2_ are the frequencies of the two waves, and *Φ*_1_ and *Φ*_2_ are the phase factors. When the two sound fields have the same frequency (*ω*_1 =_ *ω*_1 =_ *ω*), the mean square of the sound pressure at a point in space becomes

(3) $$p_{\mathrm{rms}}^{2}(r)=\frac{1}{2}\left[p_{1}^{2}(r,\omega )+p_{2}^{2}(r,\omega )+2p_{1}(r,\omega )p_{2}(r,\omega )\cos\left(\phi_{2}-\phi_{1}\right)\right]$$

If the wave reaches a certain point in space, *Φ*_1_ − *Φ*_2_ = 0 appears, which means that the phase of the soundwaves reaching this point is the same; thus,

(4) $$p_{\mathrm{rms}}(r)=p_{1\mathrm{rms}}(r,\omega )+p_{2\mathrm{rms}}(r,\omega )$$

The effective sound pressure at this point is superimposed to obtain higher sound pressure.

If a point in space is reached and *Φ*_1_ − *Φ*_2_ = 0, the soundwaves that reach the point are reversed:

(5) $$p_{\mathrm{rms}}(r)=\left|p_{1\mathrm{rms}}(r,\omega )-p_{2\mathrm{rms}}(r,\omega )\right|$$

When two soundwaves reach the r point and the same phase appears, the total effective sound pressure is the sum of the effective sound pressure of the respective soundwaves, and when the opposite phase occurs, the two soundwaves are subtracted; then, the absolute value can be measured.

According to the acoustic interference principle, if the sound pressure of two soundwaves reaching the r point is the same (ignoring the thermal viscosity loss during propagation), *p*_1_ = *p*_2_, *p* = 2*p*_1_ will occur in the same phase, forming a sonic wave belly, and when the opposite phase is reversed, *p* = *p*_1_ − *p*_2_, forming a wave node. Using this principle, we couple and superimpose two sound sources to form a sound field in this study, which has a sound pressure potential well, meaning that the sound pressure at the wave node is 0. By adjusting the phase difference, the position of the potential well changes to manipulate particles in space.

#### 2.3.2. Simulation Model

From the analysis in the previous section, it can be seen that the sound field interference principle can build a potential well and adjusting the phase difference can manipulate particles. To find similar methods suitable for standing wave acoustic field control, sound fields must be constructed. This requires the distance between the two sound sources to be an integer multiple of the half-wavelength to build a standing wave node in space. The node position and number of nodes are limited by the geometry of the acoustic device and the natural frequency of the sound device. Based on the above principles, we designed a pentagonal acoustic device. The frequency and position of the sound source change at the same time, the sound field is jointly constructed, and the soundwave node can be flexibly controlled.

**Figure 3:**
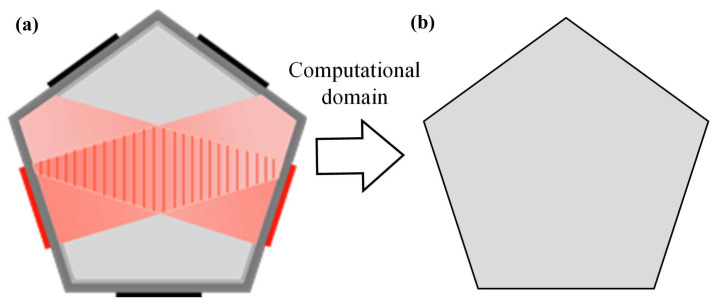
Simulation model of a pentagonal resonator: (**a**) experiment model and (**b**) numerical model.

The front view of the pentagonal acoustic device model is in Figure 3, which is a simplified model that sets the pentagon wall surface as the boundary condition of the incident sound source, the same size as the container for the actual experiment. The gray part of the model is the numerical analysis domain, and the black part refers to the chip piezoelectric ceramic. Piezoelectric ceramics excite the sound field, forming a sound field in the container to establish a sound pressure potential well.

After the model is established, the parameters of the simulation experiment need to be set. The software used for the simulation experiment is COMSOL, which is commonly used for acoustic simulations. In COMSOL, the procedure is as follows: First, the sound source is set, the edge of the pentagon is set as the displacement vibration boundary, and the normal phase is a simple harmonic vibration. Second, due to the selection of piezoelectric ceramics for the sounding device, and according to their piezoelectric strain characteristics, the vibration displacement is set to 1.97 × 10^−10^ m/s and the vibration frequency is set to 1 MHz. Finally, the model boundary in the layer basin is set to the wall boundary, and the calculation domain material is set to water.

To determine the motion state of the cell particles in the sound pressure field, the particle tracing module of the COMSOL software is coupled with an acoustic module to calculate the change in the movement state of the cell particles in water under the acoustic radiation force’s action. Although cell manipulation is simulated during the simulation, the general cell structure is composed of a three-layer structure (cell membrane–cytoplasmic–nucleus) because the simulation cannot be too complex if we want to define the simulated particles; thus, a simplified definition of the cell is required. As such, we define the cell size as 10 μm. The parameters after the simplified cell definition are shown in Table 1, as is the cell membrane decay coefficient.

**Table 1:** Cell model and parameters in simulation.

Parameter	Value
Density (kg/$m^{3}$)	1050
Diameter (μm)	10
Velocity (m/s)	1550
Attenuation coefficient (Np/m/MHz)	10.2

As the mesh of the model is divided, it needs to be parsed. Thus, the mesh size is determined according to the wavelength of the specific soundwave in the medium. During the second-order parsing process, at least five meshes are required for each wavelength length to ensure the correct solution, so that the maximum mesh size set satisfies Formula (6):

(6) $$h=\frac{l}{5}$$

where *h* is the maximum mesh size of the model and l is the wavelength. When dividing the mesh, it is necessary to pay attention to the correspondence between the wavelength of the sound source and the size of the grid. In the process of sound field simulation calculation, the calculation domain distribution of the sound field is relatively uniform, and there is no complex structure. Thus, there is no need to refine the mesh of some of the internal structures. Other simulation calculations are refined according to the calculation requirements.

#### 2.3.3. Simulation for the Formation of a Microparticle Array

After setting the basic parameters and meshing, we modify the sound source position and establish different sound fields. The goal is to verify the effect of the excitation mode in manipulating particle velocity and range.

**Figure 4:**
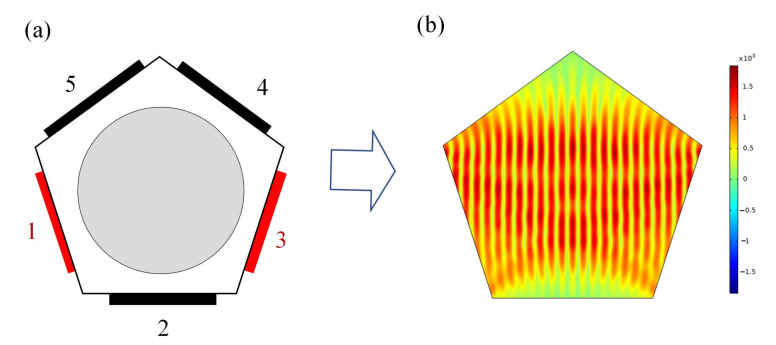
Interval excitation mode (IEM): (**a**) sound source configuration and (**b**) sound pressure distribution map.

First, the excitation sound source is set at boundaries 1 and 3 of the simulation models, as shown in Figure 4a. The parameters are frequency, 1 MHz, and sound pressure, 1 KPa. Thus, the sound field in the interval sound source excitation mode is established. After the simulation calculation, the total sound pressure distribution map (Figure 4b) is obtained, and the sound field calculation domain forms a stable sound pressure field. The two sound sources merge to form an ultrasonic node, and the sound pressure at the node position is 0 Pa.

**Figure 5:**
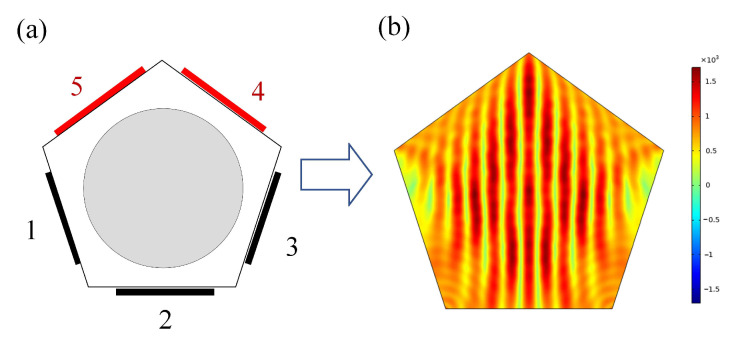
Adjacent excitation mode (AEM): (**a**) sound source configuration and (**b**) sound pressure distribution map.

Then, the sound source frequency and sound pressure of the same parameters are excited at boundaries 4 and 5, as shown in Figure 5a. The sound field in the adjacent sound source excitation mode is established, and the results (Figure 5b) are obtained after calculation. The construction mode of the two sound fields can construct the corresponding sound pressure potential well for the purposes of cell manipulation, but the sound potential well generated by the two construction methods is quite different in the distribution area of the calculation domain compared with the first sound field excitation mode. It can be seen that the direction of the sound pressure field joint line in the calculation domain has basically not changed, but the density of the sound field joint line is significantly reduced.

**Figure 6:**
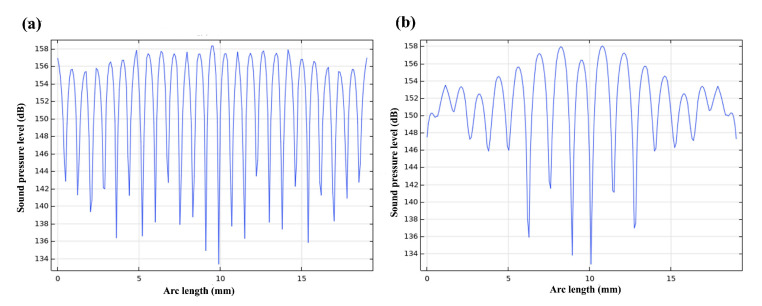
Comparison of pentagonal sound pressure level lines in the x direction in (**a**) the IEM mode and (**b**) the AEM mode.

To more intuitively see that the two modes produce potential wells in the calculation domain, the sound pressure level line plot is drawn on the horizontal diagonal of the calculation domain; the curve plot drawn is shown in Figure 6. It can be seen that the interval sound source excitation mode has more sound valleys; that is, it has more potential wells and more dense potential wells.

**Figure 7:**
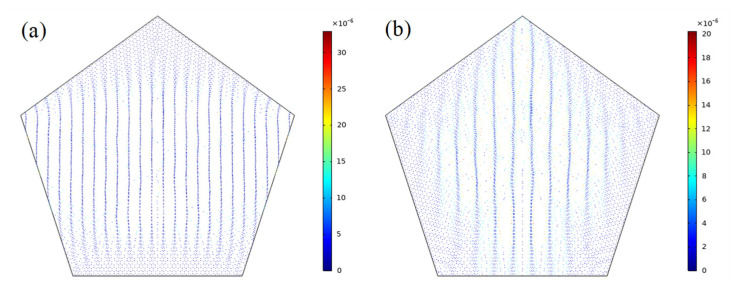
Comparison between particle manipulations in two modes: (**a**) IEM mode and (**b**) AEM mode.

The particle tracing module of the COMSOL software, coupled with the acoustic module, is applied to calculate the change in the motion state of the cell particles in the water under the acoustic radiation force’s action, as well as to observe the movement state of cell particles in two different sound source excitation modes. Figure 7 is a comparison between the two control effects, and it can be seen that the second control method has a smaller influence area.

### 2.4. Experiment for the Formation of a Microparticle Array

Regarding the study of microfluidic technology, polystyrene microspheres are often used to replace cells with positive sound source contrast factors in manipulation experiments. Therefore, this paper uses polystyrene microspheres instead of cells for manipulation experiments. The polystyrene microspheres we used are products of Tianjin Besle Co., Ltd. The diameter of the beads used in the experiment is 10 μm, the density is 1050 kg/$m^{3}$, and their size and density are comparable to the magnitude of the cells. The main parameters of the polystyrene microspheres are shown in the table below; the variation coefficient in the table indicates the uniformity of the particle size.

After the construction of the experimental, ultrasonic potential trap, particle-manipulation system, the monolayer cells are first controlled. The single-layer cell manipulation mode is divided into two excitation modes; one is the adjacent sound source excitation mode, and the other is the interval sound source excitation mode. To verify the simulation results above and to determine the most reasonable sound source excitation mode for cell manipulation, we need to change the excitation boundary position of the sound source, and connect the power amplifier to the piezoelectric ceramic at the adjacent resonator boundary and the piezoelectric ceramic at the interval boundary resonator. The voltage value is 5 V and generates a 1 MHz ultrasonic frequency. In addition, an appropriate amount of water is placed in the cavity of the pentagonal cavity to simulate the cell culture fluid, and the polystyrene microspheres are transported into the cavity using a pulsed pneumatic cell micro-delivery system. Under the microscope, the experimenter needs to adjust the magnification of the microscope to 100–1200 times and connect to the industrial camera to observe and record the entire experimental process.

The observed experimental results are then preserved, and the distance between the pressure nodes generated by the excitation is measured using a microscope. We then compare the measurements with the simulation results and observe the node density in the two excitation source modes.

## 3. Results and Discussion

### 3.1. Single-Layer Particle Pattern

**Figure 8:**
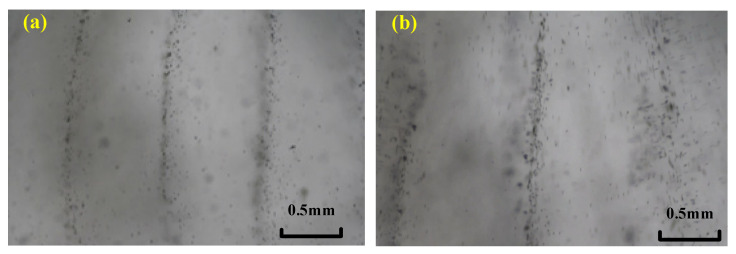
Particle pattern actuated by (**a**) the IEM mode and (**b**) the AEM mode with a driving frequency of 1 MHz.

Based on the experimental results, we can analyze the particle manipulation in the interval excitation sound source mode. As shown in Figure 8a (a particle control diagram of the interval piezoelectric ceramic sound source mode), a plurality of particles has formed in the cavity, and three columns of particles can be seen in the field of view. The distance between each line is about 0.8 mm.

Figure 8b shows a particle manipulation diagram in the excitation mode of the adjacent sound sources. In this mode, it takes 30 s for particles to form a stable particle arrangement with a line distance of 1.3 mm. At the same time, the width of the lines formed becomes 0.3 mm. However, compared with the first excitation mode, the particle arrangement is more scattered, and more particles are randomly distributed, which is due to the following reasons: First, the angle of the pentagon on both of the adjacent sides is larger; thus, the energy of the sound source is divergent, and the energy loss is large, resulting in insufficient acoustic radiation to attract particles. Second, the influence of the sound flow causes fluid flow.

In the control experiment using sound source excitation of 1 MHz, we prove that a stable particle line arrangement can be achieved in the pentagonal control device. The line spacing between the two control modes is 0.8mm and 1.3 mm, which can achieve control over the particles in two resolutions. However, the control effect in the excitation mode of the adjacent sound sources is not good: the particles formed are arranged in a wide line, and the control stability is poor. We can conclude that the stability of the particle manipulation in the excitation mode of the adjacent sound sources is low. The reason for the difference is that the acoustic flow effect is obvious in the excitation mode of the adjacent sound source, and the fluid flow causes the particulates to diffuse more.

**Figure 9:**
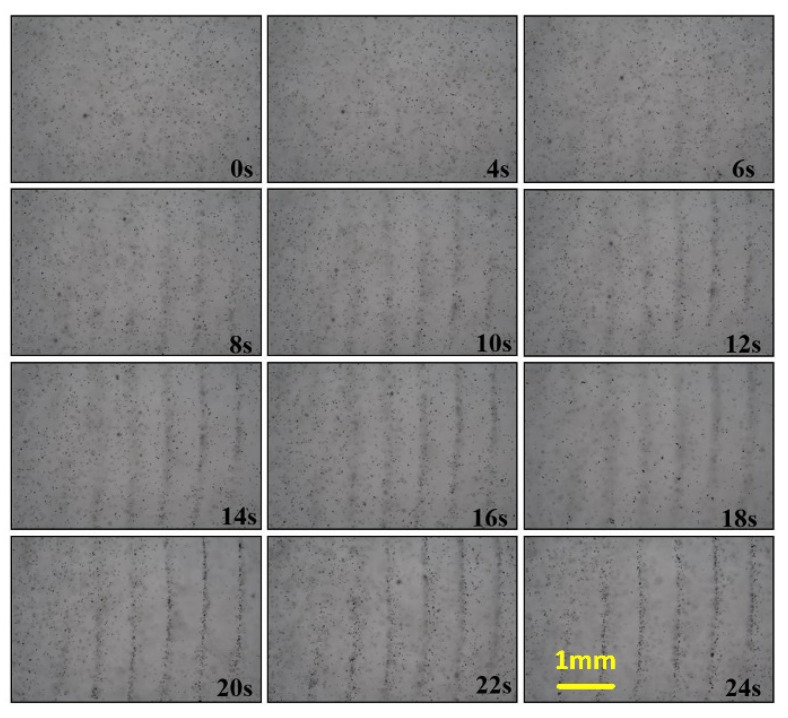
Recordings illustrating the process of particle arrangement actuated by the IEM mode with 1 MHz.

Figure 9 and Video S1 show the pattern-forming process starting from 0 s to a stable state. The frequency parameter of the piezoelectric ceramic used in the experiment is 1 MHz, and the voltage amplitude parameter is 5 V. In the figure, when the excitation time is 0 s, the particles are randomly scattered in the chamber. Piezoelectric ceramics continuously excite soundwaves, causing particles to move to the knot’s position, and at 8 s, a particle accumulation phenomenon near the knot can already be observed. At 12 s, the prototype of the arrangement of the three particles can already be seen in the field of view, but the lines formed are wider, and a stable arrangement line can be formed under the sound field’s action. When the time reaches 24 s, a stable arrangement of particles eventually forms. After 24 s, although we can see the stable particle-alignment line, there are still some particles that are free of the line. We can speculate on the reason: First, the energy at the wave node holds a limited number of particles. Second, there is a sound flow effect inside the cavity, which spreads around under the sound flow’s action, away from the position of the knot, causing some particles to be scattered around the line.

**Figure 10:**
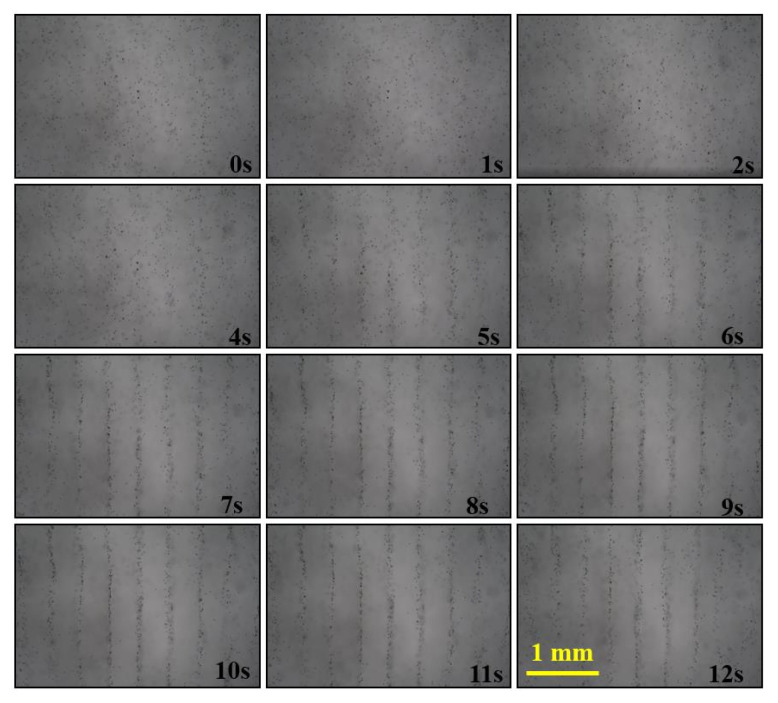
Recordings illustrating the process of particle arrangement actuated by the IEM mode with 2 MHz.

Figure 10 and Video S2 show the pattern-forming process starting from 0 s to a stable state actuated by the IEM mode with 2 MHz. The pattern becomes stable in 12 s, which is a much shorter period than with 1 MHz.

To evaluate the accuracy of the simulation, the array spacings are measured in the above experiments. A comparison of the array spacings between the simulation and experimental results is shown in Table 2. The error is about 1.25% when the power frequency is set to 1.0 MHz in the IEM excitation mode, which shows that the simulation has good accuracy.

### 3.2. Double-Layer Particle Pattern

**Figure 11:**
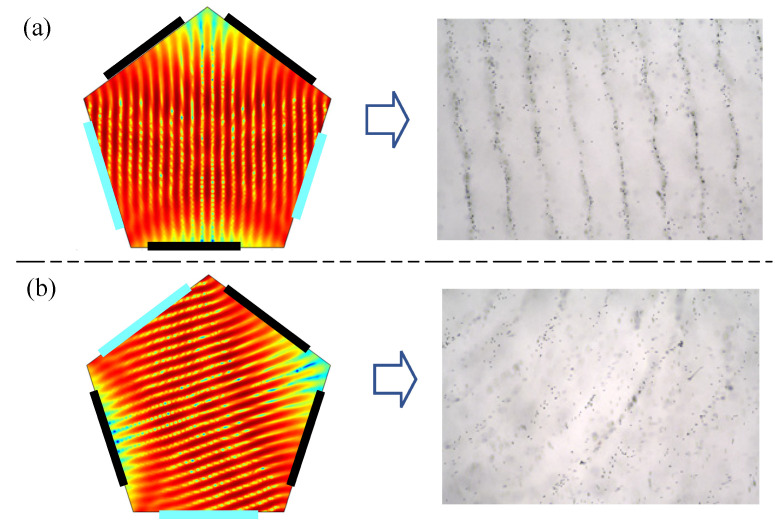
Double-layer manipulation of particles: (**a**) upper layer and (**b**) lower layer.

To form double-layer particle patterns, particles need to be manipulated in three dimensions. As shown in Figure 11 and Video S3, both depicting a multi-layer particle manipulation experiment, two planar acoustic pressure potential wells are constructed by exciting the sound source at the upper and lower intervals in a pentagonal resonant cavity. The excitation mode of the sound source is the same, but the angle of the sound potential well is about 72°. The spacing between the two layers is about 3.0 mm, and it can be seen that the three-dimensional particles can form a stable arrangement of multiple layers of particles due to particle control. The line spacing is about 0.4 mm in both layers, but the line width is 0.2 mm. Compared with the single-layer sound trap control (line spacing, 0.4 mm; width, 0.1 mm), the line spacing is not much different, but the line width increases by 1 mm. In the three-dimensional particle-control method, the influence of the sound source of the upper and lower layers leads to the formation of particles arranged in lines. These are messier. In the upper layer of the control, they form a more stable particle arrangement, but the lower layer is more affected, and it thus forms another arrangement of particles. However, this arrangement is also messier. The experimental results show that the multi-layer, acoustic pressure potential trap can assemble the particles in three dimensions, which can be used to construct a three-dimensional, multi-cell system.

## 4. Conclusions

In this paper, we presented an efficient, multi-layer microparticle pattern technique in a 3D polygon cavity using a traveling bulk acoustic wave. There are two types of excitation modes: the interval excitation mode (IEM) and the adjacent excitation mode (AEM). Theory and simulation analyses were conducted, and the results show that the above two modes can form particle arrays in the resonant cavity, which is similar to the experimental result. The line spacings in the IEM and AEM were about 0.8 mm and 1.3 mm, respectively, while the acoustic frequency was 1 MHz. Double-layer particle patterns were arrayed by a double in the resonant cavity. The spacing between the two layers was set to 3.0 mm. The line spacings were about 0.4 mm in both layers. The line width was 0.2 mm, which is larger than the single layer. Compared with standing wave manipulation, a traveling wave can be more easily modulated in real time, and no matching requirement between the size of the resonant cavity and the sound frequency is necessary. The results show that an ultrasonic traveling wave is a feasible method of manipulating particles or cells that form 3D patterns in particle–fluid flows.

## Figures and Tables

**Table 2:** Comparison of array spacings between the simulation and experimental results.

Excitation Modes	Power Frequency	Array Spacing/mm		Error
		Simulation	Experiment	
IEM	1.0 MHz	0.79	0.8	1.25%
	2.0 MHz	0.39	0.4	2.5%
AEM	1.0 MHz	1.27	1.3	2.3%
	2.0 MHz	0.64	0.57	12.3%

## Data Availability

The data presented in this study are available from the corresponding author, H.W, upon reasonable request.

## Supplementary Materials

The following supporting information can be downloaded at: https://www.mdpi.com/article/10.3390/mi13112003/s1, Video S1: Particle pattern-forming process by IEM actuating mode with frequency of 1 MHz; Video S2: Particle pattern-forming process by IEM actuating mode with frequency of 2 MHz; Video S3: Double-layer particle pattern observed by Microscope.
